# The current state of primary healthcare in Pakistan: a way forward for low-to-middle income countries

**DOI:** 10.1017/S1463423624000549

**Published:** 2024-10-31

**Authors:** Syed Hassan Ahmed, Maha Zahid, Summaiyya Waseem, Amna Zafar, Taha Gul Shaikh, Taha Sabri, Ainan Arshad

**Affiliations:** 1 Dow University of Health Sciences, Karachi, Pakistan; 2 Karachi Medical and Dental College, Karachi, Pakistan; 3 Taskeen Health Initiative, Karachi, Pakistan; 4 Department of Medicine, Aga Khan University, Karachi, Pakistan

**Keywords:** Alma-Ata, health for all, health policy, Pakistan, PHC, Primary healthcare, public health

## Abstract

**Background:**

Primary healthcare (PHC) plays a crucial role in improving health outcomes and reducing healthcare burden, especially in low-to-middle-income countries (LMICs). However, PHC has not received adequate attention in Pakistan despite its recognized importance. This study aims to examine the current state of PHC in Pakistan, identifying factors compromising its quality and effectiveness.

**Methods:**

To find relevant data, the authors conducted a thorough literature search on PubMed, Google Scholar, and Cochrane Library from inception till 2 July 2022, without any language restriction. The following keywords were employed during the literature search, separated by Boolean operators AND, OR: “Primary Healthcare”, “PHC”, “Healthcare primary”, “Primary Health”, and “Pakistan”.

**Results:**

Pakistan’s PHC infrastructure shows promise, with a considerable number of healthcare facilities in place. However, various factors hinder its effectiveness and compromise the quality of care provided. Insufficient investment, resource constraints, inadequate training of healthcare providers, lack of oversight, and limited access to essential medicines and equipment are some of the key challenges observed. Improving PHC in Pakistan is vital for addressing the population’s healthcare needs, particularly in rural areas. Adequate investment, enhanced training programs, improved oversight mechanisms, and increased availability of essential resources are necessary to strengthen the PHC system. By prioritizing PHC and addressing the identified challenges, Pakistan can enhance healthcare access, reduce healthcare burden, and improve overall health outcomes for its population.

**Conclusion:**

It is high time LMICs like Pakistan recognize PHC as the most economically feasible pathway toward accomplishing healthcare targets and adopt adequate measures to elevate its standards.

## Background

**Figure 1.**
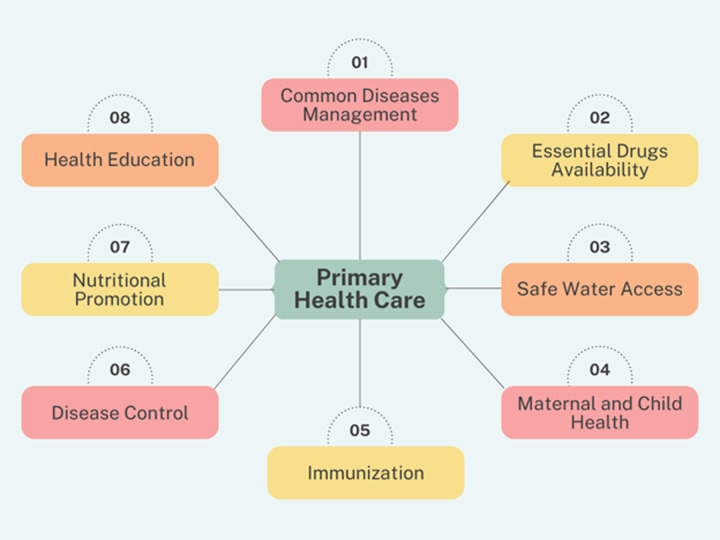
Components of primary healthcare.

The fundamental concept of primary healthcare (PHC) was first recognized as the cornerstone of “health for all policy” in 1978 at the Alma-Ata convention (Behzadifar, Taheri Mirghaed, and Aryankhesal 2017). It employs a holistic approach that emphasizes overall physical, mental, and social well-being rather than treating certain diseases and organ systems separately. According to the World Health Organization (WHO), PHC focuses on people’s needs, from primary prevention to early detection of disease and intervention, along with rehabilitative services and palliative care (Primary Health Care, 2023). It includes treatment for common illnesses and injuries, access to necessary medications, and a variety of services, such as immunization, family planning, nutrition, prenatal care, prevention, education, and the management of local epidemic outbreaks. The Alma-Ata Declaration specifically listed eight substantial components of PHC, as shown in Figure 1. The sustainable development goals (SDGs) for 2030, from goal two to goal four, include all these fundamental objectives (Behzadifar, Taheri Mirghaed, and Aryankhesal 2017).

The government, i.e., the public sector, plays a significant role in the provision and advancement of health in both developing and developed nations. This can only possibly be achieved through strong political support and the provision of financial resources. However, due to the growing complexity of healthcare systems and socioeconomic concerns, this vast network is faced with numerous challenges.

Despite having the potential to reduce the overall disease burden by a striking 70%, most countries allocate a significant proportion of their health budget toward funding secondary and tertiary health systems (Behzadifar, Taheri Mirghaed, and Aryankhesal 2017). If appropriately implemented, PHC can help low-to-middle-income countries (LMICs) save almost 60 million lives and increase life expectancy by 3.7 years (Primary Health Care, 2023).

According to the Pakistan Demographic and Health Survey (PDHS) carried out in 2017–18, only 15.4% and 5.4% of the population, respectively, have access to basic health units (BHUs) and rural health centers (RHCs) within the community. Alarming rates of 42, 62, and 74 fatalities per 1,000 live births are recorded for neonatal, infant, and under-5 mortality, respectively. Only 6 in 10 mothers and newborns receive postnatal care within two days of delivery, which is another barrier caused by limited healthcare facilities (Pakistan Demographic and Health Survey 2017-18). These difficulties are mostly caused by a dearth of healthcare professionals, especially in rural areas, where healthcare institutions are frequently understaffed, ill-equipped, and lacking in basic medications and supplies. Despite these challenges, the Government of Pakistan has launched various programs such as the National Health Vision 2016–25 to enhance the quality and accessibility of PHC services. In this review, we aim to evaluate the current status of PHC in Pakistan, highlighting its shortcomings and the way forward.

## Material and methods

To find relevant data, the authors conducted a thorough literature search on PubMed, Google Scholar, and Cochrane Library from inception till 2^nd^ July 2022, without any language restriction. The following keywords were employed during the search, separated by Boolean operators AND, OR: “Primary Healthcare”, “PHC”, “Healthcare primary”, “Primary Health”, and “Pakistan”. All relevant review studies, editorials, and correspondence were reviewed. To achieve comprehensive results, synonyms, related terms, and variant spellings were also used. Grey literature and bibliographies of the relevant articles were also screened. Articles in any language other than English were excluded. The final list of articles was generated based on the relevance to our topic.

## Results

### Structural framework and accessibility

Pakistan’s PHC system is quite extensive and comprises 600 RHCs, 5000 primary health units, 7500 first-level clinics, and over 100,000 lady caregivers working throughout the country (WHO EMRO | Primary and Secondary Health Care | Programmes | Pakistan). Despite such an extensive system, several factors compromise the quality, with lack of funds being the cornerstone. According to the preliminary federal budget 2021–2022, a total of 28.3 billion Pakistani Rupee (PKR) is set for the healthcare system, which is a mere 0.4% of the total budget, making it a clear reflection of the unsatisfactory commitment (Mirza, 2021). According to the finance ministry’s Pakistan’s budgetary expenditure report, 2019–2021, the tertiary health system remained the mainstay of funds distribution with approximately 60% allocations, while the PHC received less than 40%, particularly the mother and child health barely receiving 0.5%. Likewise, the budget for the construction of roads, highways, and bridges exceeded the healthcare allowance by striking eight-to-nine times while absolutely no funds were allocated toward population control (PRSP Budgetary Expenditures of FY 2019-20 and FY 2020-21).

Another issue pertaining to PHC is the lack of monitoring and regulation, leading to increased staff absences, long waiting times, underdeveloped facilities, inadequate maintenance, poor pharmacy practices, and so on (Hussain, Radwan, and Habib 2021). All these factors compromise the quality of PHC centers forcing people toward neighborhood doctors, which aside from costing more, poses other concerns.

The Lady Health Workers Program (LHWP), initiated in 1994, marks a significant milestone in the provision of PHC and ensures accessibility to primary, promotive, preventive, and curative services, especially in underdeveloped locations. With almost 90,000 workers catering to around 115 million women and children, it stands among the world’s most extensive community-based programs (Performance Evaluation Report - Lady Health Workers Programme in Pakistan). They serve as a liaison between the community and PHC, engaging the public in family planning, use of contraception, antenatal, natal, and postnatal care, nutritional and diet modifications, educational awareness to overcome emerging or endemic diseases, improving sanitation, extended program on immunization (EPI), preventing and treating prevalent diseases such as malaria and tuberculosis, polio vaccination, and other needs of time like currently SARS-CoV-2 prevention, management, and vaccination. Despite having beneficial outcomes and enormous potential to elevate PHC standards, particularly in rural and underdeveloped localities, the LHWP experiences a series of major structural obstacles: inadequate funding alongside the delayed release of funds resulting in supply and facilities shortfalls; incoordination between LHWs and local hospitals; recruitment deficiencies resulting from underpays, largely distanced commute without adequate transportation facilities, over-assigning of tasks; and lack of regular training sessions (Performance Evaluation Report - Lady Health Workers Programme in Pakistan). Moreover, the public’s hesitancy toward immunization, contraceptives, family planning, and natal care further hinders their tasks.

### Essential drug availability

The provision of essential medications is one of the eight elements of PHC. The WHO coined the concept of essential medicines and encouraged countries to ensure their uniform accessibility in proper dosages with assured quality and affordability for local communities. Poor pharmaceutical supply and distribution networks are among the many issues limiting access to medications in underdeveloped nations like Pakistan (Dixit *et al.,* 2011).

The pharmaceutical industry in Pakistan is worth more than Rs.300 billion and is growing at a 12% yearly rate. The pharmaceutical business makes an annual contribution of about 1% to Pakistan’s GDP (Pharmaceutical Industry, 2018). Despite this, Pakistan still has a substantial accessibility gap for essential medications. The overall essential drug storage at PHC, according to Hussain *et al*., fell short of WHO requirements, with limited access to necessary medications for chronic illnesses and concerning storage conditions in storerooms and dispensing areas (Hussain, Radwan, and Habib 2021). No medication was found to have a high availability rate in a study analyzing the accessibility of specific important medications in Baluchistan, and several were inaccessible at public health facilities, in contrast to the private sector. Similarly, certain medicines like ciprofloxacin and clarithromycin were unaffordable and exceeded the average daily wage (Bibi *et al.,* 2022).

The absence of these medications at public healthcare units prompts the public to purchase necessary medications at a higher cost from private pharmacies. Furthermore, a lack of proper regulation by the relevant drug authorities culminates in sales without prescription, retailers providing drugs at higher costs, poor storage facilities, provision of falsified drugs, and illegal smuggling. In such cases, the unfavorable compensating approaches utilized as stand-ins for missing EMs could cause nontreatment, subpar therapy, and a high likelihood of prescription mistakes (Rafi *et al.,* 2021).

### Maternal and child health

Compared to many similar low-resource nations, Pakistan has one of the worst pregnancy-related outcomes in the world (Aziz *et al.,* 2020). While there has been some improvement over the past few years, it still drastically lacks relative to the set standards, culminating in a high maternal mortality rate (MMR), pregnancy-related complications, and under-five infant mortality (Bhutta and Hafeez 2015). Between 2010 and 2018, the MMR was recorded at 314 per 100,000 births compared to only 124 in other LMICs. Moreover, the neonatal mortality rate (NMR) stood at 49.4 per 1,000 live births against an average of 20.4 in others. Similarly, stillbirths, preterm delivery, and low birth weights were also more prevalent. Likewise, according to NMR data from 2016, Pakistan ranked as the highest birth risk country, surpassing the Central African Republic, Afghanistan, and Somalia. In Pakistan, one in every 22 infants born in 2016 passed before the end of the first month. Moreover, due to the lack of a proper surveillance system, these figures are, at best, estimations, and the actual numbers may be much worse (Aziz *et al.,* 2020). Several factors contribute to these worrisome findings, including funds scarcity, underdeveloped facilities, high illiteracy among females of reproductive age, low engagement in family planning, and religious and cultural barriers.

### Safe water supply

Another integral component of PHC involves providing safe, clean drinking water, which can significantly enhance population health by reducing and even eradicating numerous preventable diseases. Pakistan has an adequate water supply, yet these resources are under a great deal of strain as a consequence of industrialization, urbanization, rapid population expansion, sewerage leakage, and natural catastrophes, particularly floods. Furthermore, inadequate water disinfection, quality monitoring, and lagging sewerage systems contribute significantly to the high waterborne disease burden. The current population in Pakistan is estimated to be 225 million, and it is projected to surpass 250 million by 2025. This increase in population is placing an added strain on the country’s water resources, which have been rapidly depleting over time. In fact, per capita availability of water has fallen from 5,000m^3^ in 1951 to 1,100m^3^ in 2005, and this is expected to decrease further to 800m^3^ by 2025. Additionally, Pakistan’s water demand is increasing at an average annual rate of 10% (Ishaque *et al*., 2023). Research studies on community health link poor drinking water quality to 40% of deaths. Urban water supplies do not match the WHO’s drinking water standards, making safe drinking water accessible only to 20% of the population, with the remaining 80% compelled to consume unsafe water (Daud *et al.,* 2017).

Among South-Asian countries, Pakistan had the highest rate of typhoid, with 493.5 cases per 100,000 people in 2018 (Daud *et al.,* 2017). In Pakistan’s pediatric population, hepatitis A accounts for 50–60% of cases of acute viral hepatitis (A. S. Butt and Sharif 2016). Similarly, cholera is an endemic illness with a history of several outbreaks. A recent spike in cholera cases has been documented in Karachi wherein, between January and April 2022, 129 confirmed cases of cholera were reported (S. H. Ahmed *et al.,* 2022). Moreover, the aquatic amoeba Naegleria fowleri, which causes primary amoebic meningoencephalitis (PAM), has also been documented in Karachi’s domestic water supply and is a growing health concern. The first PAM case was reported in 2008, and as of October 2019, 146 cases have been documented (Ali *et al.*, 2020). In Pakistan, where 25 million people continue to defecate in the open, coliform bacteria heavily contaminate water supplies, hence hastening the spread of illnesses (Malnutrition Crisis - Pakistan | ReliefWeb 2018).

A survey carried out by the Pakistan Council of Research on available water resources in 23 major cities of Pakistan revealed four major contaminants predominating in water sources, with bacteria accounting for the majority (69%). Following these were fluoride (5%), nitrate (14%), and arsenic (24%) (Daud *et al.,* 2017). Unfortunately, drinking water quality issues receive little attention, and the relevant authorities continue to prioritize other concerns.

### Nutritional promotion

Despite being an agricultural country, the Pakistani population experiences a significant level of food insecurity. Over the past 40 years, malnutrition, a significant public health challenge, has been purposefully neglected, as shown by the recent spate of pediatric fatalities in Thar, Sindh. With stunting costing the nation 3% of its GDP annually, Pakistan is experiencing one of the greatest malnutrition crises in the world (Malnutrition Crisis - Pakistan | ReliefWeb 2018). Given the current disregard and massive flooding, reaching the SDG-2 objective of nutrition and food security by 2030 seems practically unfeasible. According to estimates from the Food and Agriculture Organization (FAO), Pakistan reports a 20.3% prevalence of undernourishment (PoU) (Pakistan Overview of Food Security and Nutrition: Improving Access to Food - 2019 - 2020). Due to rapid population growth, increasing food insecurity, and water shortages, this percentage is predicted to worsen in the upcoming years (Malnutrition Challenge 2022).

The National Nutrition Survey (NNS) 2018 depicted that, among the under-five age group, four of every 10 children in Pakistan are stunted, while 17.7% suffer from wasting. More than half (53.7%) of the children were anemic. Moreover, 18.6% lack adequate zinc, and about 51.5% have vitamin A deficiency. Similarly, Vitamin D deficiency affects nearly 62.7% of this age group. Nearly one in eight adolescent females and one in five adolescent males are underweight, with anemia affecting over half of the adolescent girls (56.6%). Among women of reproductive age, one in seven (14.4%) women are under-nourished, with approximately 41.7% being anemic, 79.7% lacking sufficient vitamin D, and Vitamin A deficiency affecting more than a quarter (27.3%) (Final Key Findings Report 2019).

### Immunization

Due to their ability to prevent significant morbidity and mortality, immunization is regarded as one of the most effective and economically advantageous public health interventions (M. Butt *et al.,* 2020). Properly implemented immunization programs can be immensely beneficial in achieving health-related goals outlined in United Nations SDG-3, which urges governments to eradicate all preventable infectious causes of under-five mortality by 2030 The Aga Khan University 2023.

Unfortunately, the extent of under-immunization in Pakistan is concerning. With the country falling short of the immunization goals set by the international community, Pakistan experiences significant mortality from vaccine-preventable diseases (VPDs) despite vaccines being affordable and easily administered (M. Butt *et al.,* 2020). The Expanded Programme of Immunization (EPI), which was launched in 1978, aims to protect the population against fatal VPDs, including Tuberculosis, Poliomyelitis, Diphtheria, Pertussis, Tetanus, Hepatitis B, Haemophilus influenzae type B, Pneumonia, Meningitis, Rotavirus diarrhea, Typhoid, Measles, and Rubella. Since its inception, this program has faced multiple challenges with manqué of planning, supervision, and surveillance; logistical shortcomings; public’s hesitancy; the impact of religious community leaders, political meddling and mistrust seeded by armed conflict; natural disasters, and the ensuing internal displacement of huge communities being a few of them (M. Butt *et al.,* 2020).

Pakistan has some of the highest infant and child death rates in the world as a result of low immunization rates. In comparison to a global average of 8%, the country’s under-five population, which makes up about 15% of the population, accounts for 50% of deaths (M. Butt *et al.,* 2020). Pakistan’s district variation is an unusual phenomenon. This is important because the majority of Pakistan’s 164 million residents (70%) do not have access to immunizations since they live in rural regions. Furthermore, the 6000 fixed immunization centers are disproportionally spread out across the nation (M. Butt *et al.,* 2020). According to the Third-Party Verification Immunization Coverage Survey (TPVICS) conducted in Pakistan between July 2020 and December 2020, 76.1% of children under 2 years of age have received a complete series of immunizations as advised by the country’s government. Among the remaining 17.7%, more than one in every six children has only received vaccinations partially, while 5.9% never received any (AKU Releases Findings of Pakistan’s Largest Vaccine Survey).

In regard to communicable illnesses, Pakistan’s inability to eradicate polio significantly adds to its negative reputation, and it remains one of the only two countries still endemic to polio (Endemic Countries – GPEI). Pakistan had the highest number of poliomyelitis cases in 2011 globally, endangering the Global Polio Eradication Initiative (Owais *et al.,* 2013). There were 359 polio cases documented in 2014, with 306 reported in Pakistan. In certain regions, immunization rates against polio are as low as 50% (M. Butt *et al.,* 2020). Similarly, 147 cases were documented in 2019. As of September 2022, 17 verified cases have been reported, compared to just one occurrence in 2021 (Polio Cases Update 2020 | Across Pakistan U2019s Provinces ; GPEI-Pakistan). In addition to the obstacles mentioned previously, polio vaccination coverage has been low due to violence against vaccine providers. Additionally, even after the initial dose administration, subsequent doses are less likely to be taken, which makes vaccine programs ineffective at reaching their objective (M. Butt *et al.,* 2020).

### Disease control

Pakistan’s disease control strategies have been commendable, particularly in containing Polio transmission. Despite being one of the two remaining countries battling Polio, Pakistan has taken several steps to eradicate the disease, including using acute flaccid paralysis (AFP) surveillance. Pakistan has the largest environmental surveillance network in the world, with 65 sites located strategically across the country. The minimum global standard for non-polio AFP cases is two per 100,000 population below 15 years, which Pakistan has exceeded by 6.8 in 2015 to more than 13.4 in June 2021. In 2020, Pakistan reported a 43% reduction in WPV1 cases from the previous year, and as of August 25, 2021, only one WPV1 case had been reported (Mbaeyi *et al.,* 2021; History of Polio Vaccination).

Furthermore, the Polio Eradication Initiative (PEI) and surveillance system implemented in Pakistan had also helped the country combat COVID-19. The PEI team supported Pakistan’s response to the pandemic by organizing and realigning roles and responsibilities at the National Emergency Operations Centre. They also strengthened surveillance, infection prevention and control, contact tracing, and developed new data systems to track diseases. Risk communication and community engagement efforts were also enhanced to raise awareness about the disease. However, there is a dire need for such strong surveillance of other infectious diseases in the country (PEI-Contribution-to-COVID19). For LMICs such as Pakistan, almost 50% of reported deaths are attributable to infectious diseases that can be readily avoided via appropriate surveillance and prevention initiatives. Each year almost 27,000 tuberculosis cases, 300,000 cases of malaria, and 2.5 million cases of hepatitis B are reported. Similarly, 47,120 dengue fever cases were documented in 2020. Additionally, contagious diseases like measles and influenza are quite prevalent, with 8,345 measles cases in 2019 and 192 cases in 2018 being recorded, respectively. Typhoid was identified in 22,571 people between 2016 and 2020. The incidence rate of HIV, which was 0.12 in 2019, exhibits a concerning rising trend (Bilal *et al.,* 2022). Thus, a robust surveillance system is required to enable the country to take appropriate preventive measures and determine their effectiveness.

To improve surveillance, measures are being taken at both the provincial and federal levels. In 2006, a Field Epidemiology and Laboratory Training program was introduced, which monitors multiple diseases via nationwide response units. Similarly, the Disease Surveillance System established in Punjab collects data from province-wide healthcare facilities and issues weekly bulletins. Contribution from this system helped prevent a measles outbreak in 2012 and an influenza epidemic in 2015. Currently, the system monitors data for 26 communicable diseases and is being upgraded to cover a broader range (Shaikh *et al.,* 2022). The Disease Early Warning System in earthquake-affected areas was established in 2005 by the WHO and the Ministry of Health, Pakistan. Later, in 2005 and 2009, this technique was employed to cover flood and conflict-affected areas in northern Pakistan, respectively (Shaikh *et al.,* 2022). While the work is appreciable, lack of funding and logistical support remains the main obstacle to developing an efficient nationwide surveillance system. However, with the help of international collaboration, Pakistan can replicate currently used systems in noncovered regions and devise a nationwide monitoring system.

### Treatment of common diseases and injuries

Noncommunicable diseases (NCDs) have major detrimental impacts on both the economy and society. It is worrisome that their burden continues to increase, particularly among vulnerable LMICs, as a result of global industrial growth and sedentary lifestyle changes (Almas *et al.,* 2022).

In Pakistan, NCDs cause more than 50% of adult deaths (Almas *et al.,* 2022). Currently, mortality from NCDs considerably surpasses that from communicable diseases. Data for regional trends show that the disease burden in South Asia, including Pakistan, shifted from communicable diseases in the 1990s, including diarrhea and lower respiratory tract infections, to noncommunicable diseases in 2010, including ischemic heart disease (IHD) and chronic obstructive pulmonary disease (Jafar *et al.,* 2013). The four NCDs that contribute to the highest NCD mortality in Pakistan include cancer, cardiovascular illnesses (CVD), chronic respiratory diseases, and diabetes (Almas *et al.,* 2022).

According to the WHO country profile (2014), there were 25.3% of Pakistanis with elevated blood pressure, 19 % with CVD, 3% with diabetes, 6% with chronic obstructive pulmonary disease, 8% with cancer, 23% smoked, and 0.1% consumed alcohol (Naseem *et al.,* 2016). A cross-sectional study conducted in an urban settlement of Lahore in 2018–2019 showed that of all the respondents, 40.1% had hypertension, 15.8% had diabetes, and 17.0% had IHD (Kazmi *et al.,* 2022). Another community-based cross-sectional survey covering 1210 households demonstrated about 38.7% of individuals had hypertension or IHD, 34.4% had oro-dental health problems, 24.3% were physically disabled, and 14.6% had diabetes (Naseem *et al.,* 2016). According to projections made by Jafar *et al*., using population-level death rates, 3.87 million Pakistanis will die from NCDs such as cancer, chronic respiratory illnesses, and cardiovascular diseases between 2010 and 2025. Additionally, the predicted financial cost of NCD mortality will increase from $152 million in 2010 to $296 million in 2025 (Kazmi *et al.,* 2022).

In Pakistan, a national action plan (NAP) was created in 2004 to address the rising burden of NCDs, which was complemented with a follow-up plan in 2014. The NAP was an extensive plan that utilized a coordinated approach, multisectoral action, and public–private collaboration. The government initially demonstrated a strong level of devotion, but it gradually withdrew that support (Almas *et al.,* 2022; Jafar *et al.,* 2013). To minimize NCDs on a national level, an integrated approach encompassing supervision, primary care interventions, education, and monitoring is now required. The insufficient budgetary funds set aside for the primary and secondary prevention of NCDs serve as additional proof of the rising instability (Almas *et al.,* 2022).

The estimated annual cost of all mental diseases in Pakistan was PKR 81,922 (USD 1394.65) for hospitalizations and PKR 19,592 (USD 333.54) for outpatient care. Mental health concerns are also a major problem in Pakistan. This burden may be alleviated by incorporating mental health services in PHC, which may ease early identification and intervention, hence reducing the overall psychiatric disease burden (Malik and Khan 2016). It is estimated that if PHC adequately plays its role in the management of mental illnesses in Pakistan, it could potentially result in substantial economic savings, amounting to USD 1577.19 million in total (Malik and Khan 2016).

### Health education

Improved health outcomes necessitate a certain degree of health education. Pakistan has not yet fully explored the idea of health education (W. Ahmed *et al.,* 2018). The country still struggles with low health literacy, frequently leading to delayed consultation, poor medication adherence, and a lack of comprehension of wellness and disease prevention (Sabzwari 2017). In 2015, a cross-sectional survey conducted in Karachi showed that 82.4% of the population had poor health literacy, with 70% having trouble accessing and comprehending health information for personal well-being (W. Ahmed *et al.,* 2018). Therefore, enhancing healthcare awareness may have a significant impact on the well-being of our population. A practical approach for conveying healthcare information is to employ rapidly developing smartphone apps and media.

## Discussion

Pakistan features a centralized healthcare system in which the Federal government has authority over key health decisions. Thus, a communication vacuum exists between the federal, provincial, and district levels. However, despite all the challenges faced by the public sector, the country has formulated multiple strategies and implemented plans to improve the healthcare system (Kurji *et al.*, 2016).

In 2000, Pakistan became a signatory body for the eight Millennium Development Goals (MDGs) adopted in 2000 by the United Nations (UN) members for the improvement of human life and health conditions. Additionally, the government began work on the Medium-Term Development Framework (MTDF) 2005–10 and Vision 2030, which also includes health and nutrition. To work toward achieving the MDGs, the Pakistani government was the first in the world to establish a nationwide public–private partnership. Another important development in Pakistan’s health sector is the country’s effort to reduce maternal and newborn mortality with financial assistance from foreign donors. As a result, the Pakistani government created vertical programs with the help of foreign aid, such as the Maternal and Child Health Care Program, the EPI, the Oral Rehydration Salt Packets Information, Education, and Communication (IEC) Campaign, and the National Maternal and Child Health (MCH) Program at all levels of the healthcare system (Kurji *et al.*, 2016).

The Sehat Sahulat Program (SSP), a revolutionary healthcare initiative, was launched by the government of Pakistan in 2015 to achieve the goals of universal health coverage (UHC) as set by WHO. The program was first introduced in Khyber Pakhtunkhwa (KP) province. Under this initiative, the ‘Sehat Insaf Cards’ were issued to families throughout the province. The SSP’s principal objective was to grant over 4 billion people in all 35 districts of the KP province access to basic health services. Under this plan, individuals can receive up to Rs. 1 million ($6,000) in annual treatment in more than 400 public and private health institutions across the KP, which accounts for around 25% of the province’s total health facilities (Hasan *et al.,* 2022).

The program has rapidly expanded throughout Punjab province. On 9 December 2020, the Government of Punjab allocated Rs.65 billion to implement SSP with plans to provide a Sehat Insaf Card to families in all 36 districts throughout Punjab. Unfortunately, the governments of Sindh and Baluchistan did not provide provincial funding for the UHC initiatives, which made it difficult to provide UHC to the 60 million residents of those two provinces, who make up about 30% of Pakistan’s population. Additionally, on December 28, 2020, the Pakistani Prime Minister (PM) stated that the SSP program would now be accessible to residents of Azad Jammu and Kashmir (AJK) in more than 350 hospitals that qualified; the expansion was anticipated to help more than a million households. Overall, more than 27 million families have registered with the program as of March 8, 2022, across various provinces (Hasan *et al.,* 2022).

While the new Sehat Insaf Card program is commendable, it needs to be managed efficiently. For a developing country like Pakistan, political determination and financial stability are the key concerns. The UHC will cost roughly 400 billion PKR, or about 62%, of Pakistan’s 650 billion PKR annual national health budget. As a result, efficient allocation of resources, planning, and a reliable source of funding from national and international organizations are necessary. Additionally, for its fair execution, UHC’s linkage with National Database Organization (NADRA) and Health Management Information System (HMIS) is crucial. Moreover, as mentioned above, the disparities in distribution throughout the nation need to be dealt with to ensure that everyone in the community has access to healthcare. Additionally, Since UHC is a relatively new concept in Pakistan, public awareness efforts are required to inform individuals about the process of getting enrolled (Farooq *et al.*, 2022).

Amidst the shortcomings of the conventional PHC framework, it has enormous potential to ameliorate the overall healthcare system in Pakistan. According to a study conducted in Khyber Pakhtunkhwa, the tertiary care unit cannot surpass more than 20% of human resource capacity, leading to its poor performance and unpreparedness for emergencies and disasters (Haroon and Thaver 2022). Strategic and organizational modifications may not only alleviate the healthcare standards but also reduce the burden on secondary and tertiary resources. Many problems currently experienced stem from the paucity of funds due to disproportionate budgetary allocations. The WHO recommends that countries assign at least 1% of their gross domestic product (GDP) to PHC. Not only this but efficient usage and instant availability must also be ensured.

Furthermore, collaborations with international and local nonprofit organizations can help to escalate funds availability without burdening the country’s economic standings. With almost two-thirds population residing in rural areas, establishing primary health clinics and hospitals will cost a fortune; however, improving the LHWP can not only make PHC accessible but also ensure a more cost-effective approach. Most maternal and under-five mortality reports are from these areas (Performance Evaluation Report - Lady Health Workers Programme in Pakistan), so the LHWP can significantly help reduce the incidences. Recognition, logistics availability, routine training, upscaling salaries and target-oriented bonuses, strategic recruitment cycles, a structural hierarchy with adequate hiring, and timely evaluation can help overcome the existing loopholes, alongside serving as a source of motivation for many.

Despite an extensive PHC system comprising multitudinous clinics and health units, the relevant authorities must ensure strict regulation and routine evaluation of recruited staff, medicines availability, storage conditions, infrastructure, and efficient funds usage. Measures need to be taken to promote family medicine perception among medical and nonmedical populations, alongside establishing more family medicine postgraduate training centers. The General Physician practice can be adopted from the United Kingdom’s model, where family medicine practitioners hold significant credit for ranking the UK’s health system as one of the best compared to other healthcare systems (Razai and Majeed 2022). Similarly, the government needs to set up a centrally monitored system incorporating both public healthcare centers and private neighborhood clinics to ensure internationally followed standards, prevention of malpractices, and improved disease surveillance. Moreover, safe drinking water must be made accessible, and vaccination programs should be promoted along with establishing a well-devised nationwide surveillance system to reduce the overall disease burden. While up-stepping secondary and tertiary healthcare is equally essential, a well-devised and implemented PHC system can help provide immediate results without requiring massive investments and logistics.

## Conclusion

Pakistan confronts immense obstacles in keeping up a well-monitored PHC system, despite having great potential to tackle the nation’s healthcare challenges. Its quality is compromised by a lack of resources, poor oversight, lengthy wait times, underdeveloped facilities, and subpar pharmacy environments, which force the populace to consult insufficiently skilled independent practitioners. Furthermore, similar deficiencies are also present in the highly capable LHWP. The COVID-19 pandemic highlighted Pakistan’s appalling lack of healthcare facilities, staff, and supplies. Therefore, it is high time LMICs, like Pakistan, recognize PHC as the most economically suitable pathway toward accomplishing healthcare targets and adopt adequate measures to elevate its standards.

## Data Availability

This study used publicly available data.
